# Highly Biocompatible Apigenin-Loaded Silk Fibroin Nanospheres: Preparation, Characterization, and Anti-Breast-Cancer Activity

**DOI:** 10.3390/polym15010023

**Published:** 2022-12-21

**Authors:** Weikun Qu, Peng Ji, Xibin Han, Xianglong Wang, Yang Li, Jin Liu

**Affiliations:** 1 Department of Oncology, The Second Hospital of Dalian Medical University, Dalian 116023, China; 2 College of Pharmacy and Chemistry & Chemical Engineering, Taizhou University, Taizhou 225300, China; 3 Laboratory Animal Center, Jinzhou Medical University, Jinzhou 225300, China; 4 Department of Cardiovasclar Medicine, The Second Hospital of Dalian Medical University, Dalian 116023, China; 5 Department of Nephrology, The Second Hospital of Dalian Medical University, Dalian 116023, China

**Keywords:** silk fibroin, apigenin, pharmacokinetics, anti-breast-cancer

## Abstract

Breast cancer is among the most common fatal diseases among women. Low-toxicity apigenin (AGN) is of interest due to its good antitumor activity, but its clinical application is severely limited due to its poor water solubility and low bioavailability. An effective strategy to enhance the anti-breast-cancer activity of AGN is to develop it as a nanodelivery system. Silk fibroin (SF) is an ideal drug carrier with good biocompatibility, biodegradability, and a simple extraction process. This paper develops a novel and efficient apigenin-loaded silk fibroin nanodelivery system (SF-AGN) by nanoprecipitation with SF as a carrier. The system was characterized in terms of morphology, zeta potential, particle size, ultraviolet (UV), infrared (IR), and synchronous thermal analyses (TG-DSC), and the in vitro cytotoxicity and in vivo pharmacokinetics were examined. Finally, the chronic toxicity of SF-AGN in mice was studied. The SF-AGN nanodelivery system has good dispersibility, a hydrated particle size of 163.35 nm, a zeta potential of −18.5 mV, an average drug loading of 6.20%, and good thermal stability. MTT studies showed that SF-AGN significantly enhanced the inhibitory effect of AGN on 4T1 and MDA-MB-231 cells. Pharmacokinetic studies have demonstrated that SF-AGN can dramatically improve the bioavailability of AGN. The results of toxicity experiments showed that SF-AGN is biocompatible and does not alter normal tissues or organs. In sum, the SF-AGN nanodelivery system is a promising drug-delivery system for the clinical treatment of breast cancer.

## 1. Introduction

Globally, cancer is a major public health problem; it is a disease in which certain cells of the body grow uncontrollably and spread to other parts of the body, and it has high morbidity and mortality [1]. Breast cancer (BC) is the leading cause of cancer-related disability-adjusted life years and death in women worldwide, with 2.1 million new cases diagnosed globally each year. It is estimated that 627,000 deaths occurred in 2018, accounting for 15% of the annual cancer deaths among women [2]. Breast cancer is clinically characterized by high incidence, high invasiveness, and poor prognosis. The most effective treatment for early-stage breast cancer patients is surgery combined with other adjuvant treatments. As for breast cancer patients who are clinically diagnosed with cancer spread or recurrence, especially those in an advanced stage, chemotherapy is usually used to significantly prolong the survival time of patients [3]. Unfortunately, chemotherapy is usually associated with adverse effects such as side effects and drug resistance as well as long treatment cycles and high treatment costs [4]. Compared with other medications, plant-derived compounds and secondary metabolites have low side effects and are attractive drugs for breast cancer treatment [5].

Apigenin (AGN) is a natural flavonoid compound widely found in various fruits, vegetables, and herbs, with the chemical name 4′, 5, 7-trihydroxyflavonoids. AGN has been used for centuries as a traditional natural medicine due to its various antibacterial, anti-inflammatory, anticancer, and antiviral activities [6]. Recently, AGN has received much attention because of its good anticancer activity and low toxicity. It has been reported that AGN significantly inhibits various tumor cells, including breast cancer, cervical cancer, renal cancer, and liver cancer. [7,8]. Its mechanism may be related to inhibiting tumor cell migration or invasion, promoting cell cycle arrest, inducing cell apoptosis, and interfering with tumor signaling pathways [9]. Although AGN is an effective anticancer molecule with reasonable safety and no adverse metabolic reactions and is classified as a class II drug, it has disadvantages such as poor water solubility, a short half-life, and relatively low bioavailability, which significantly limits its further clinical application [10,11]. Therefore, it is necessary to improve the solubility and bioavailability of AGN to achieve better antitumor effects.

With the rapid advances in nanotechnology and materials science, a variety of novel nanocarrier materials have opened up new opportunities for disease treatment. Active pharmaceutical ingredients (API) can be loaded into nanocarriers and delivered to target sites (e.g., tumors) through enhanced permeability and retention (EPR) effects [12,13]. Silk fibroin (SF), a natural polymeric fibrous protein extracted from silk, has been recognized as a biomaterial by the U.S. Food and Drug Administration (FDA). SF has good biocompatibility, degradability, and regenerability, as well as structural adjustment, and when used as a drug carrier, it can enhance the controlled release of drugs, reduce the side effects and the number of drug administrations [14,15,16]. Therefore, it is a promising candidate to be developed for the preparation of new drug delivery systems. Interestingly, SF has the ability to bind to various types of compounds such as small molecules (e.g., paclitaxel), proteins, peptides, and nucleic acids [15]. This is due to the amphiphilic nature of SF molecules, which have hydrophilic amino acid residues (e.g., aspartic acid, Figure S2) and hydrophobic amino acid residues (e.g., glycine) and thus can bind hydrophilic drugs via van der Waals forces and hydrophobic drugs via hydrophobic interactions and p–p stacking [17].

This study is based on the functions of natural polymer SF as a carrier material for drug delivery, with good biocompatibility and biodegradability, controlled drug release, reduced adverse effects, and improved therapeutic efficacy. SF nanospheres were developed and used to load AGN, the prepared AGN-loaded silk fibroin nanospheres (SF-AGN) were characterized, and their morphology and thermal stability were evaluated. In addition, breast cancer cell lines were selected, and the in vitro cytotoxicity of SF-AGN was assessed. Next, the pharmacokinetic behavior and toxicity of the SF-AGN nanospheres were evaluated at the animal level.

## 2. Materials and Methods

### 2.1. Materials

Apigenin, lithium bromide, potassium bromide, and heparin sodium were purchased from Shanghai McLean Biochemical Technology Co., Ltd., (Shanghai, China). Mulberry silk fibroin was purchased from Huzhou Xintiansi Biotechnology Co., Ltd., (Huzhou, China). MTT Assay kit was purchased from China Biyuntian Biotechnology Co., Ltd., (Shanghai, China). Dipotassium hydrogen phosphate, potassium dihydrogen phosphate, and dimethyl sulfoxide were purchased from Shanghai Sinopharm Chemical Reagent Co., Ltd., (Shanghai, China). Methanol (HPLC grade) was purchased from China Xilong Chemical Co., Ltd., (Guangzhou, China). Mice (4–5 weeks old) were from the Animal Experiment Center of Jinzhou Medical University, China (Jinzhou, China). The animal study protocol was approved by the Animal Ethics Committee of Jinzhou Medical University of China (20220311001). All the water used was double-distilled water.

### 2.2. Extraction of Silk Fibroin

**Figure 1:**
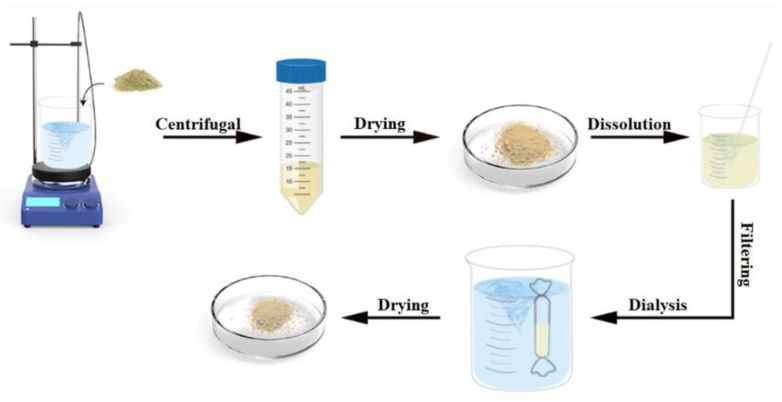
Diagram of silk fibroin extraction.

Figure 1 shows the process of SF extraction from cocoons [18,19]. A solution of Na_2_CO_3_ at a concentration of 0.02 M was prepared accurately, and the cocoons were boiled in Na_2_CO_3_ for 30 min. This process was repeated three times, replacing the solution with fresh Na_2_CO_3_. Next, the solution was centrifuged at 3000 rpm for 10 min, the supernatant was discarded, the degummed silk fibers were rinsed with distilled water, and the excess water was squeezed. The silk fibers were then placed in an oven and dried at 50 °C to obtain the silk fibers. The extracted filament fibers were dissolved in Ajisawa reagent (CaCl_2_: ethanol: water, 1:2:8 molar ratio) at 60 °C for 4 h. The insoluble impurities were removed by filtration; the fibers were transferred to a dialysis bag with a cut-off molecular weight of 14,000 Da for 48 h using deionized water as the dialysis medium. The retention solution was transferred to a beaker and dried in an oven to obtain SF. The retained solution was transferred to a beaker and dried in an oven (50 °C) to obtain a lumpy solid, which was gently ground to obtain SF flowable powder.

### 2.3. Preparation of SF-AGN Nanospheres

Hydrophobic amino acid residues in SF molecules, such as tyrosine, glycine, and alanine, can effectively allow the entrapment of hydrophobic drug AGN through hydrophobic interaction and p–p stacking [17]. SF nanospheres were prepared by the nanoprecipitation method. Briefly, an appropriate amount of AGN was weighed and dissolved in DMSO. The resulting solution was added dropwise to SF aqueous solution (2 mg/mL), stirred and mixed well and then left for 4 h in a refrigerator at 4 °C and stirred for 12 h in a water bath at 40 °C; the solution was centrifuged at 3000 r/min for 30 min, and the precipitate was washed three times with purified water and dried in a vacuum drying oven at 40 °C, and SF-AGN nanospheres were obtained.

### 2.4. Characterization of SF and SF-AGN Nanospheres

#### 2.4.1. Analysis of the Secondary Structure of SF

The secondary structure analysis was carried out by scanning the infrared spectra of silk fibroin. A small amount of silk fibroin was taken, mixed well with potassium bromide in proportion, and pressed into a translucent sheet of 1 mm thickness by a press. The infrared spectrum of silk fibroin was obtained by Fourier infrared spectroscopy with wave number 4000–400 cm^−1^ and a resolution of 4 cm^−1^.

#### 2.4.2. Particle Size, Zeta Potential, and Surface Morphology of SF-AGN

The SF-AGN nanospheres were diluted with a small amount of water to create a suspension of 0.2 mg/mL concentration. Their particle size and potential were determined using a particle size analyzer (Brookhaven 90Plus PALS, Suffolk, NY, USA). The morphological characteristics of SF-AGN were investigated using transmission electron microscopy (TEM, FEI Talos F200X, FEI Corporation, Hillsboro, OR, USA). Sample preparation for TEM was performed with a 0.2 mg/mL suspension of nanospheres in pure water followed by sonication for 3 min with an amplitude of 30% [20]. A drop of this suspension was placed on a 200-mesh copper mesh coated with carbon. After natural drying at room temperature, the copper mesh was stained with a 1% phosphotungstic acid solution for 60 s. The copper mesh was imaged by TEM at a voltage of 80 kV.

#### 2.4.3. Drug Loading (DL) of SF-AGN Nanospheres

The content of AGN was determined by UV-visible spectrophotometry [21]. The standard curve obtained is y = 0.0218x + 0.009, R^2^ = 0.9992 (Figure S3). The amount of SF-AGN was weighed precisely, added to 30% ethanol-DMSO mixture, sonicated to make it fully dissolved, filtered using 0.22 μm microporous membrane, and its absorbance was measured by UV to calculate its AGN content and the drug loading of SF-AGN; the experiment was repeated three times. The DL was calculated using the following formula:

DL (%) = (Mass of drug in NPs)/(Mass of NPs) × 100%

#### 2.4.4. Fourier Transform Infrared Spectroscopy (FTIR)

SF-AGN was taken appropriately, mixed with potassium bromide in proportion, and then pressed. The transmittance spectra of the silk fibroins were measured by FTIR spectroscopy (WQF-510A, Beijing Ruili, Beijing, China) with a wave number of 4000–400 cm^−1^ and a resolution of 4 cm^−1^. Another appropriate amount of SF blank carrier, AGN, SF, and AGN mixture was taken and analyzed by infrared spectroscopy according to the above conditions.

#### 2.4.5. Thermal Stability Test of SF-AGN

The SF-AGN nanospheres were weighed appropriately and tested by a simultaneous thermal analyzer (TGA, TGA/DSC Type 3+, Mettler-Toledo, Greifensee, Switzerland). The heating rate was set to 10 °C/min, the heating range was 25–400 °C, and the controlled N_2_ flow rate was 50 mL/min.

### 2.5. Cell Experiments

#### 2.5.1. Cell Culture

Two cell models, the human breast cancer cell line (MDA-MB-231) and murine breast cancer cell line (4T1), were selected for this experiment. The MDA-MB-231 was grown according to the culture conditions recommended in the literature [22]. MDA-MB-231 was cultured using DMEM medium containing 10% fetal bovine serum in a 37 °C, 5% CO_2_ incubator. Fresh medium was changed twice a week and passaged at 1:3, with trypsin digestion at room temperature for 1–2 min. 4T1 was grown using RPMI 1640 medium containing 10% fetal bovine serum in a 37 °C, 5% CO_2_ incubator. The medium was changed every 2–3 days and passaged at 1:4 at 80% confluence.

#### 2.5.2. In Vitro Antitumor Activity

Cells were seeded into 96-well plates at a density of 10^5^ cells/well and incubated in 95% air and 5% CO_2_ incubator at 37 °C for 24 h. Then, different concentrations (3.75–60 μg/mL) of AGN and SF-AGN nanospheres were added to the plates, and after 24 h of treatment, cells were washed twice with PBS, and serum-free medium without phenol red was replaced in all wells. Next, 5 mg/mL MTT solution was added to incubate the cells for 4 h. The MTT solution was discarded and washed twice with PBS, and 100 μL of DMSO was added and shaken to dissolve the formazan fully. The optical density (OD) of each well was measured at 490 nm using an enzyme marker, and the cell survival rate was calculated [cell survival rate (%) = OD administration group/OD blank control group × 100%]. Cell viability was expressed as the percentage of viable cells compared to the control group. A growth medium without the treatment drug was used as a control in each experiment.

### 2.6. Animal Experiments

#### 2.6.1. Chromatographic Conditions

The column used in this experiment was a C18 column (4.6 × 250 mm, 5 μm) [23]. The mobile phase was acetonitrile-0.2% phosphoric acid aqueous solution (50:50) at a flow rate of 1 mL/min, the detection wavelength was 270 nm, the column temperature was 30 °C, isocratic elution was used, and the injection volume was 20 μL each time.

#### 2.6.2. Plasma Processing

Briefly, 100 microliters of plasma was taken in a 1.5 mL centrifuge tube, to which 1 mL of methanol was added to precipitate the protein. The plasma proteins were precipitated by vortex mixing for 1 min, followed by centrifugation at 12,000 rpm for 3 min. The supernatant was passed through a 0.22 μm microporous membrane, and the drug concentration was calculated according to the above chromatographic conditions.

#### 2.6.3. Dosing and Sampling Protocols

Six mice were randomly divided into two groups of three mice each and fasted for 12 h before the experiment but were allowed to drink freely. Blood was collected by orbital blood sampling at the 1st, 2nd, 3rd, 4th, 5th, 6th, and 7th hours after administration, and 0.1 mL of blood was placed in a centrifuge tube with sodium heparin solution, treated in the same way as above, and stored in a refrigerator at −80 °C. An equal amount of saline was also injected intraperitoneally to maintain a constant blood volume in the animals.

### 2.7. In Vivo Toxicity Study

To assess the chronic toxicity of SF-AGN, mice were treated with SF-AGN for two weeks at a dose of 25 mg/kg. After 2 weeks, the mice were sacrificed, and various organs (heart, liver, spleen, lungs, and kidneys) were removed by autopsy and fixed in 4% paraformaldehyde. These tissues were sectioned, stained with hematoxylin-eosin (H&E), and photographed and observed [24].

### 2.8. Statistical Analysis

Microsoft Excel calculated the mean and standard deviation (SD). SD analysis was performed on all samples in triplicate. Statistical analysis was performed by one-way analysis of variance (ANOVA) followed by Bonferroni’s post hoc comparison test. A confidence level of *p* < 0.05 was considered significant.

## 3. Results and Discussion

### 3.1. Characterization of SF and SF-AGN Nanospheres

#### 3.1.1. Analysis of the Secondary Structure of SF

**Figure 2:**
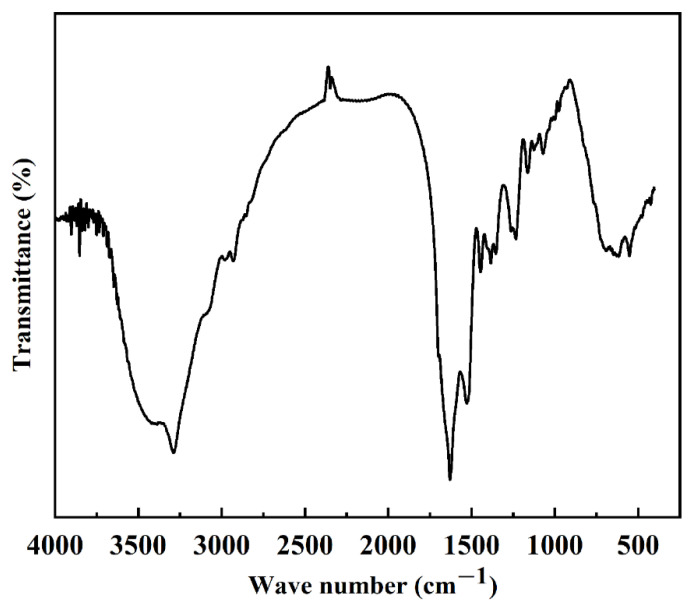
Infrared absorbance spectrum of silk fibroin.

Protein secondary structure is the structure of a polypeptide chain formed by hydrogen bonding and coiling, including α-helix, β-folding, β-turning, and irregular coiling. When the external conditions change, the protein secondary structure changes accordingly. Currently, Fourier infrared spectroscopy is the main method to study protein secondary structure. According to the literature, the secondary structure of the silk fibroin is different, and the wave numbers of the characteristic peaks corresponding to the amide bonds in its molecular structure are also different. The main secondary structure in the structure can be judged [25,26]. The wave numbers of the characteristic peaks corresponding to the amide bonds in the molecular structure are shown in Table 1. According to the results in Figure 2 and Table 1, it can be found that the FTIR spectra of SF exhibit typical peaks of crystalline β-sheet at 1530 cm^−1^ (amide II), 1625 cm^−1^ (amide-I), and 1263 cm^−1^ (amide-III), demonstrating that SF was in its stable conformation [17]. The secondary structure of the extracted silk protein in this experiment was mainly β-folded, and its crystal structure was a more stable type, which was consistent with the previously reported results [17,27] and indicated that we successfully extracted SF.

#### 3.1.2. Particle Size, Zeta Potential, Surface Morphology, and DL of SF-AGN

**Figure 3:**
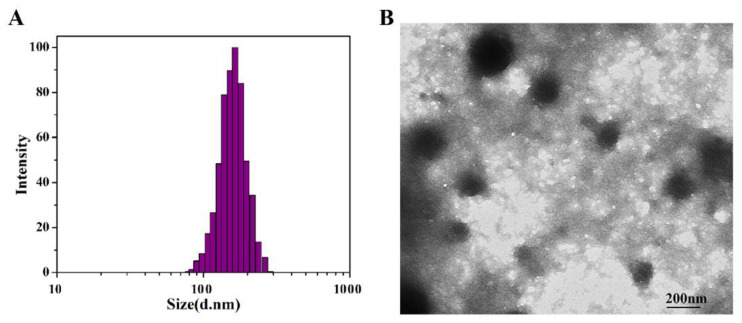
(**A**) Size distribution based on the intensity of SF-AGN and (**B**) transmission electron micrographs of SF-AGN nanospheres (magnification ×10,000).

The DLS particle size analysis of SF-AGN nanospheres is shown in Figure 3A. The particle size of SF-AGN is uniformly distributed with a “bell-shaped curve”, and the average particle size is 163.35 nm ±1.22 nm. The morphology of SF-AGN nanospheres is shown in Figure 3B. It can be seen that the nanospheres are oval or spherical in shape, the particle size is mainly distributed around 140 nm, and no apparent adhesion was observed. This result is slightly smaller than the DLS result, which may be because the particles were dry in TEM. In contrast, under DLS conditions, on the one hand, the particles were dispersed in water, and the swelling effect was caused by protein–water interaction; on the other hand, the DLS size included the diffusion layer around the particles [25]. The zeta potential of the nanospheres was also measured to be −18.5 mV. Zeta potential is a crucial determinant of colloidal dispersion stability. Zeta potential describes the degree of electrostatic repulsion between neighboring, similarly charged particles [28]. The zeta potential of SF-AGN nanospheres was greater than 15 mV in absolute value, indicating good colloidal stability [27]. The UV test results showed that the drug loading capacity of SF was 6.20% ± 0.25%, which could meet the needs of subsequent formulation studies.

#### 3.1.3. FTIR of SF-AGN

**Figure 4:**
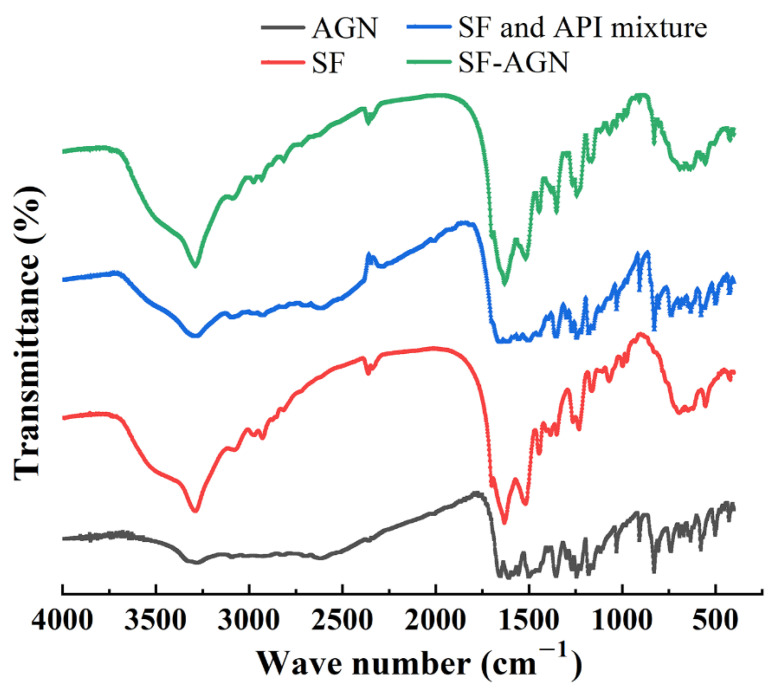
Fourier transform infrared spectroscopy.

The results of IR analysis of the SF blank carrier, AGN, SF, AGN mixture, and SF-AGN are shown in Figure 4. From the figure, it can be seen that SF-AGN has characteristic peaks at 1630 cm^−1^, 1517 cm^−1^, 1243 cm^−1^, and 691 cm^−1^, from which it can be inferred that the secondary structure of SF nanospheres is still dominated by β-folding after loading AGN, indicating that the loading of AGN does not affect the secondary structure of SF and still has specific stability. Meanwhile, compared with SF nanospheres, new characteristic absorption peaks appeared at 2815 cm^−1^, 2715 cm^−1^, 1350 cm^−1^, and 829 cm^−1^, which corresponded to the absorption peaks of AGN, indicating that AGN was successfully loaded.

#### 3.1.4. Thermal Stability of SF-AGN

**Figure 5:**
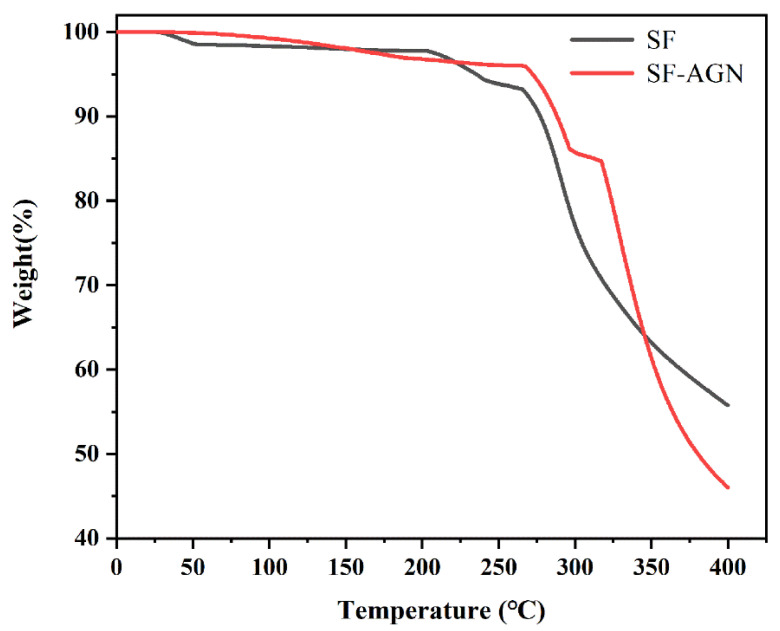
The thermogravimetric curve of SF and SF-AGN.

The thermogravimetric curves of SF-AGN nanospheres are shown in Figure 5. It can be learned from the figure that when the temperature was less than 260 °C, the weight loss rate of SF and SF-AGN was about 5%; their thermal stability was good and the difference was not obvious, which was mainly caused by the loss of adsorbed water and structural water on the surface and in the pore channel of SF nanospheres by heat [29], and the degradation of macromolecules did not occur. Furthermore, when the temperature rises to the range of 260–400 °C, the weight loss rates of SF and SF-AGN were about 40% and 50%, respectively, and the rate of mass loss began to accelerate, and the amount of loss increased, indicating that the organic matter and macromolecules in the nanospheres began to decompose in this temperature interval. When the temperature was 400 °C, the residual mass of SF was 55%, while the residual mass of SF-AGN was 45%, mainly because when the temperature was greater than 300 °C, the AGN in SF-AGN also underwent thermal decomposition, resulting in a rapid weight percentage decrease, and the weight loss was eventually higher than that of SF. In summary, SF-AGN nanospheres have good thermal stability.

### 3.2. In Vitro Cytotoxicity

**Figure 6:**
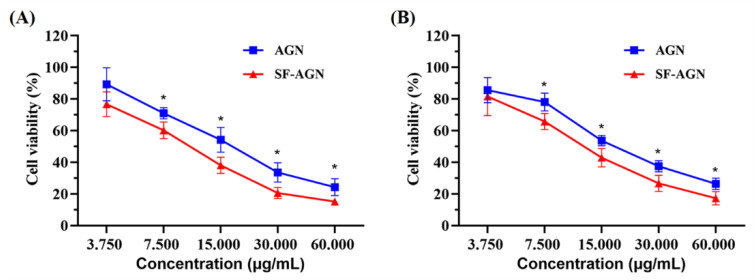
(**A**) Cell viability of AGN and SF-AGN with 4T1 cells after 24 h incubation; (**B**) AGN and SF-AGN with MDA-MB-231 cells after 24 h incubation. *: *p* < 0.05 vs. AGN.

To assess the cytotoxicity of free AGN and SF-AGN, MTT assays were performed using breast cancer cell lines (4T1 and MDA-MB-231). Cells were incubated with serial concentrations (3.75–60 μg/mL) of free AGN or SF-AGN for 24 h. Both free AGN and SF-AGN inhibited 4T1 (Figure 6A) and MDA-MB-231 (Figure 6B) breast cancer cells in a concentration-dependent manner. However, SF-AGN showed better cytotoxic effects on both breast cancer cell lines compared to free AGN in the concentration range of 7.5 to 60 μg/mL (*p* < 0.05 vs. AGN), suggesting that encapsulation of AGN in SF enhances the inhibition of AGN on breast cancer cells in vitro and therefore that SF-AGN nanoparticles have sound anti-breast-cancer effects. These results are consistent with previous reports highlighting the increased toxicity of common drugs when encapsulated in nanoparticle systems by a mechanism that may be attributed to the preferential uptake of nanoparticle carriers followed by efficient drug release within tumor cells [27,30].

### 3.3. Pharmacokinetic Studies

**Figure 7:**
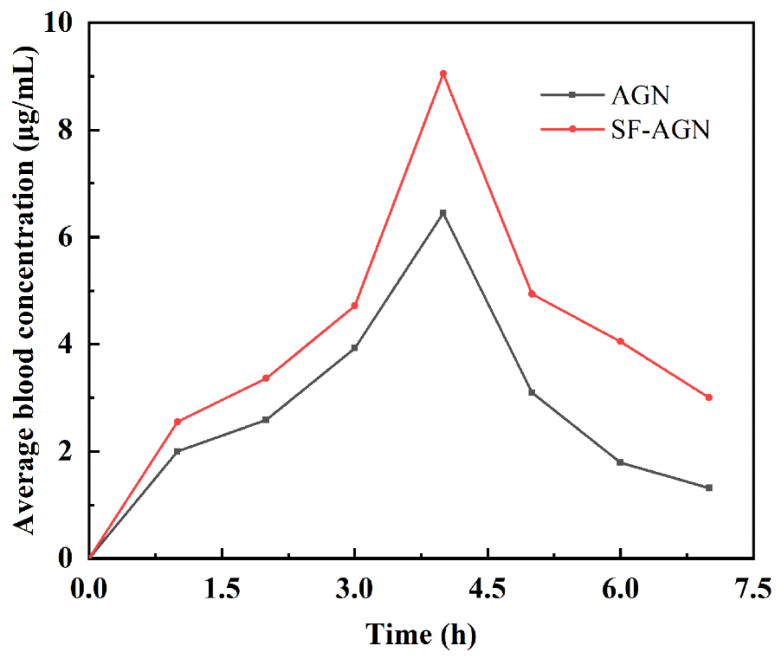
Drug–time curves of AGN and SF-AGN.

Using HPLC (UV230II, Dalian Elite Analytical Instruments, China) analysis, the AGN chromatogram is shown in Figure S1, and the results show that the peak time of AGN was about 6.15 min with good peak shape. The blood concentration of the samples was calculated, and the mean blood concentration–time curve was plotted using the mean blood concentration as the vertical coordinate and the sampling time as the horizontal coordinate (Figure 7). From the drug–time curves, it can be seen that the blood concentration of each period of the SF-AGN group was significantly higher than that of the AGN group, indicating that SF-AGN nanospheres can be absorbed into the blood faster and the metabolism rate in the body is slowed down, which can improve the bioavailability of AGN.

Relevant pharmacokinetic parameters were calculated using the PK Slover software, and the non-atrial model non-pulsatile delivery mode was chosen for analysis. The pharmacokinetic parameters are shown in Table 2. Compared with free AGN, SF-AGN effectively prolonged the half-life and increased the relative bioavailability by 1.47 times, while the C_max_, AUC_0-t_, and MRT_t_ were all raised to different degrees. The improved bioavailability may be attributed to the direct uptake of SF through the cell membrane by the lymphatic system, which facilitates drug absorption [31].

### 3.4. In Vivo Toxicity Study

**Figure 8:**
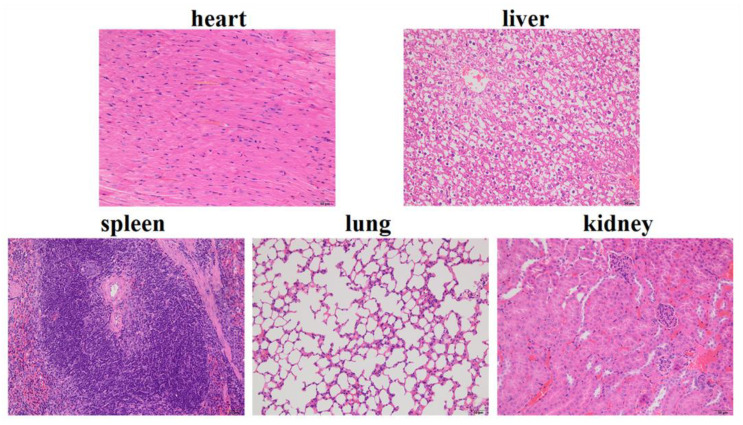
Images of H&E-stained major organ tissue sections of SF-AGN.

We evaluated the biosafety of SF-AGN, as this property is a crucial parameter for using nanotherapeutics in cancer treatment [32]. Histological analysis (H&E staining) of major organs (heart, liver, spleen, lung, and kidney) showed negligible damage in the SF-AGN treatment group (Figure 8). No histopathological changes were found in the major organs. Cardiac myocytes were clear and well-arranged, and no necrosis, hemorrhage, or inflammatory exudates were observed. Therefore, the above results suggest that SF-AGN has a perfect safety profile after administration [24].

## 4. Conclusions

In this study, the nanoprecipitation method was successfully used to prepare an apigenin-loaded silk fibroin nanodelivery system (SF-AGN) with silk fibroin nanospheres as drug carriers. It exhibited nanoscale size (163.35 nm), spherical or oval shape, good thermal stability, and high drug loading. MTT experiments showed that SF-AGN showed significantly increased cytotoxicity against MDA-MB-231 and 4T1 cells compared to AGN. In vivo pharmacokinetic studies revealed that SF-AGN significantly prolonged the half-life of AGN and improved its bioavailability compared to free AGN. In addition, in vivo toxicity experiments showed that SF-AGN had good biocompatibility and no organ toxicity in mice. The above results suggest that SF could be a promising nanocarrier, and SF-AGN may be an effective agent for the treatment of breast cancer.

## Figures and Tables

**Table 1:** Infrared characteristic peaks and secondary structure relationship table.

Secondary Structure	Characteristic Peak I (cm^−1^)	Characteristic Peak II (cm^−1^)	Characteristic Peak III (cm^−1^)	Characteristic Peak IV (cm^−1^)
α-Screw	1650–1655	1540–1555	1235	650–670
β-Fold	1615–1635	1525–1541	1260	690–700
Random crimp	1635–1648	1510–1525	1235	650

**Table 2:** Pharmacokinetic parameters of AGN and SF-AGN.

Parameters	Unit	AGN Groups	SF-AGN Groups
t_1/2_	h	1.51	2.93
C_max_	μg/mL	9.80	10.84
C_last_	μg/mL	1.31	3.00
AUC_0-t_	μg/mL × h	20.50	30.18
MRT_t_	h	3.81	4.06
Cl/F	(μg)/(μg/mL)/h	27.25	11.50

## Data Availability

Not applicable.

## Supplementary Materials

The following supporting information can be downloaded at: https://www.mdpi.com/article/10.3390/polym15010023/s1, Figure S1: High performance liquid chromatogram of apigenin; Figure S2: Chemical structure of silk fibroin; Figure S3: The obtain standard curve.
