# Supplementary material for: Highly Biocompatible Apigenin-Loaded Silk Fibroin Nanospheres: Preparation, Characterization, and Anti-Breast-Cancer Activity
Source: Polymers (Basel). 2022 Dec 21;15(1):23. doi: 10.3390/polym15010023 (PMC9823476; doi:10.3390/polym15010023)
Supplement: Supplementary file 1 [file polymers-15-00023-s001.zip › polymers-2070156-supplementary.pdf]

Supplementary Materials

Highly Biocompatible Apigenin-loaded Silk Fibroin Nanospheres: Preparation, Characterization, and Anti-Breast-Cancer Activity

Weikun Qu [1], Peng Ji [2], Xibin Han [3], Xianglong Wang [2], Yang Li [4,*] and Jin Liu [5,*]

[1] Department of Oncology, The Second Hospital of Dalian Medical University, 116023 Dalian, China
[2] College of Pharmacy and Chemistry & Chemical Engineering, Taizhou University, 225300 Taizhou, China
[3] Laboratory Animal Center, Jinzhou Medical University, 225300 Jinzhou, China
[4] Department of Cardiovasclar Medicine, The Second Hospital of Dalian Medical University, 116023 Dalian, China
[5] Department of Nephrology, The Second Hospital of Dalian Medical University, 116023 Dalian, China
* Correspondence: liyang0901@dmu.edu.cn (Y.L.); liujin900119@dmu.edu.cn (J.L.)

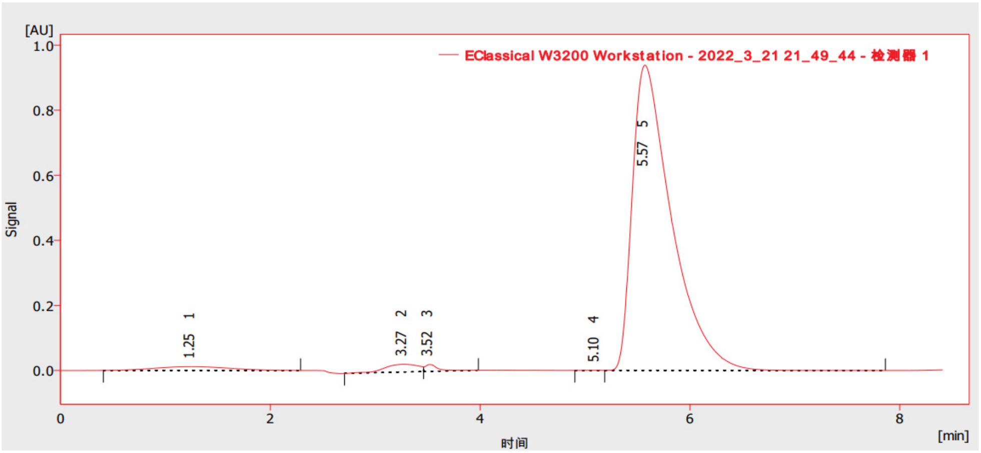

Figure S1. High performance liquid chromatogram of apigenin.

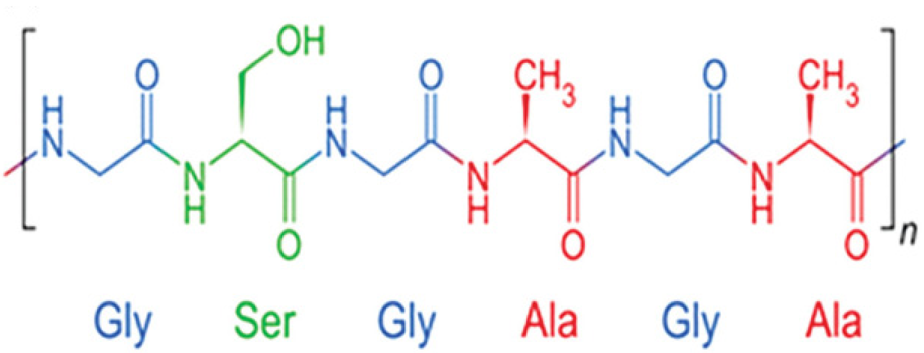

Figure S2. Chemical structure of silk fibroin.

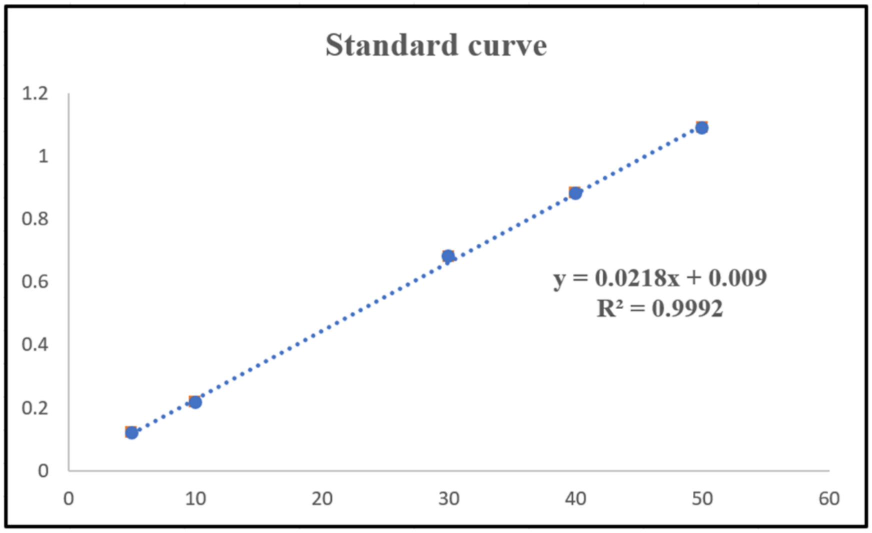

Figure S3. The obtain standard curve.
